# The association of dietary acid load (DAL) with estimated skeletal muscle mass and bone mineral content: a cross-sectional study

**DOI:** 10.1186/s40795-022-00658-w

**Published:** 2023-02-14

**Authors:** Fatemeh Gholami, Niki Bahrampour, Mahsa Samadi, Niloufar Rasaei, Habib Yarizadeh, Sina Naghshi, Khadijeh Mirzaei

**Affiliations:** 1 Department of Community Nutrition, School of Nutritional Sciences and Dietetics, Tehran University of Medical Sciences (TUMS), P.O Box 6446, Tehran, 14155 Iran; 2 Department of Nutrition, Science and Research Branch, Islamic Azad University (SRBIAU), Tehran, Iran

**Keywords:** Muscle, Bone density, Obesity, Dietary acid load, Net endogenous acid production, Potential renal acid load

## Abstract

**Background & Aims:**

Dietary patterns that promote mild metabolic acidosis may have a negative effect on bone and muscle, and a high dietary acid load (DAL) may be detrimental to skeletal muscle mass and bone mineral content. However, the association between skeletal muscle mass and bone mineral content with dietary acid load has not been consistently reported in previous studies. The objective of the study was to evaluate the association of potential renal net acid load (PRAL) and net endogenous acid production (NEAP) with bone mineral content and skeletal muscle mass in pre-menopause women with overweight or obesity in Iran.

**Method:**

Three hundred and ninety women with a body mass index (BMI) of 25 were included in this cross-sectional study. We used a validated 147-item semi-quantitative food frequency questionnaire (FFQ) for evaluating the dietary intake. Based on the dietary data, potential renal net acid load (PRAL) and net endogenous acid production (NEAP) were calculated. Muscle mass and bone mineral content were estimated by a bioelectrical impedance analyzer (BIA).

**Results:**

After controlling for potential confounders, we discovered a significant linear relationship between PRAL (β = -0.027, 95%CI = -0.049 to -0.004, *P* = 0.02) and NEAP (β = -0.05, 95%CI = -0.097 to -0.003, *P* = 0.03) and skeletal muscle mass index. However, there was no significant difference between SMM and BMC across PRAL and NEAP tertiles.

**Conclusion:**

PRAL and NEAP were found to be inversely related to skeletal muscle mass index among overweight/obese women. Further research is required to establish whether this relationship is important for musculoskeletal health in these populations.

## Introduction

Muscle mass decline and bone mineral loss are significant public health issues in our aging population and can lead to muscle weakness, greater numbers of falls and fractures, fall-related injuries, hospitalization and early death [1–6]. The prevalence of sarcopenia, characterized by a decline in muscle mass and function, has been estimated at 9.9–40.4% among adults in the community [7, 8].

Apart from aging, accumulating evidence has demonstrated the associations between musculoskeletal health and diet composition as well as acid–base balance [9]. In nutritional epidemiology, dietary acid load (DAL) in human diets has been explained by both net endogenous acid production (NEAP) and potential renal acid load (PRAL) [10].

Generally, foods with acid-generating capacity, such as meat, cheese, eggs, and grain products, lead to an increased dietary net acid load because of their sulfur and phosphate content [11, 12]. Oxidation of the sulphur-containing amino acids, methionine and cysteine content of them is involved in the formation of hydrogen ions, which subsequently promotes systemic acidity [12]. Bone, as a primary buffer system, is critical to correcting acid–base imbalances by releasing alkaline salts [13, 14]. It should be noted that calcium is one of the most important components of bone mineral content (BMC) and is essential for ensuring bone health [15]. Thus, long-term exposure to net acid-producing diets could increase bone alkali. The elevated bone alkali is bound to minerals (including calcium) to counteract the acidic environment. As a result, calcium loss, dissolution of the bone mineral content and a consequent decline in bone mineral density (BMD) will occur and consequently make it susceptible to fracturing [12, 16–19].

It is well established that persistent metabolic acidosis may stimulate the impairment of skeletal muscle function by decreasing muscle protein synthesis and increasing proteolysis [20]. With muscle breakdown, amino acids can be used for hepatic synthesis of glutamine and, in turn, ammonia in the kidney. In later stages, ammonia accepts protons and ammonium ions are excreted [21]. Thus, skeletal muscle mitigates the acidosis to maintain acid–base balance. As a consequence, higher acid production will result in more muscle mass decline [22–25]. In addition, there is a positive relationship between muscle mass and bone density, according to the mechanical forces of muscle on bones. So, it has an important role in preventing falls and fractures [26–34].

The importance of dietary acid load (DAL) in skeletal muscle mass and bone mineral content is still being debated. Some observational studies in children and adults have concluded that there are inverse associations between dietary acid load and bone mass [35–41]. For instance, in NHANES data from 1218 men > 60 y old, higher PRAL was associated with lower femoral BMD [42]. In another cohort study of German children aged 6–18 y, inverse associations between dietary PRAL and bone mineral content were observed [35, 39]. Furthermore, PRAL was found to have a negative association with musculoskeletal health in middle-aged to older men and women [43]. It is known that mild metabolic acidosis has been proposed for the loss of skeletal muscle [25, 44, 45]. However, some studies have reported no relation between bone mineral density (BMD) and PRAL and/or NEAP [23, 46, 47]. Of note is that, women in comparison to men are highly influenced by the negative impact of DAL on total lean mass [23] and differences in muscle strength and bone diseases are identified between obese and non-obese persons [48, 49]. Therefore, according to these findings and because of no population studies which have assessed the effects of dietary acid load (PRAL/NEAP) on skeletal muscle mass as well as bone mineral content in high-risk groups such as overweight and obese women, we aimed to evaluate the associations between dietary acid load with bone mineral content (BMC) and skeletal muscle mass among Iranian pre-menopause women with overweight or obesity aged 18–64 years.

## Materials and methods

### Study population

**Fig. 1:**
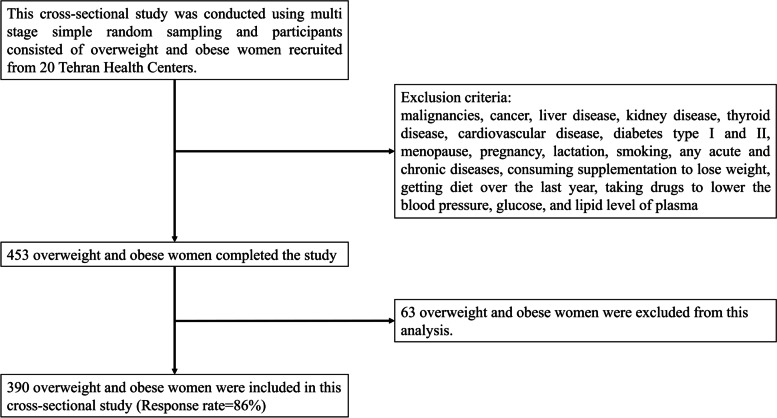
Flow chart of subjects’ enrollment

This cross-sectional study was conducted using multi stage simple random sampling and participants consisted of 390 women were recruited in 2018 from January to February. 453 women were invited to participate from 20 Tehran Health Centers. Indeed 20 health centers were randomly selected from all health centers of the Tehran University of medical sciences. People who were referred to Tehran health centers and met the inclusion criteria were randomly selected to participate in the study. 453 overweight and obese women completed the study. 63 overweight and obese women were excluded from this analysis and 390 overweight and obese women were included in this cross-sectional study. So that response rate was 86% (Fig. 1). Adult women between the ages of 18 and 64 who had a body mass index (BMI) of 25 or above were eligible. All malignancies, cancer, liver disease, kidney disease, thyroid disease, cardiovascular disease, diabetes type I and II, menopause, pregnancy, lactation, smoking, any acute or chronic diseases, taking weight-loss supplements, going on a diet in the previous year, and taking drugs to lower blood pressure, glucose, and lipid levels in plasma were all exclusion criteria. All participants in the study signed a written informed consent from that was provided before the start of the study. Also, the present study and informed consent were approved by the local ethics committee of the Tehran University of Medical Sciences (TUMS), Tehran, Iran with ethics number IR.TUMS.MEDICINE.REC.1399.636.

### Assessment of body composition and anthropometric analysis

To assess the body composition of all participants, we used a bioelectrical impedance analyzer (BIA) (Inbody 770 Co., Seoul, Korea) by following the techniques, procedures, and precaution in the manufacturer’s protocol [50]. Participants stand on the balance scale while grasping the BIA handles in bare feet. Indeed, the BIA calculated the various measures measurements by passing an electric signal that flows through the palms and soles of the feet. Participants take off any metal items, their shoes, and extra clothes. It takes 15–20 s to check the body composition and weight, skeletal muscle mass, fat-free mass, fat mass, visceral fat, body fat percentage, bone mineral content and limb skeletal muscle mass.

#### Assessment of anthropometric indices

We measured the body weight of participants without shoes and with a minimum of clothes by using a calibrated digital scale to the nearest 100 g. We measured the participants' heights with a non-elastic tape, with a precision of less than 0.5 cm, while they were in normal condition and standing beside the wall. For measuring the BMI, we divide the weight (in kilograms) into height squared (in square meters). Overweight is defined as BMI 25–29.9 kg/m2 by the World Health Organization, and obesity grades 1, 2, and 3 are defined as BMI 30–34.9 kg/m2, BMI 35–39.9 kg/m2, and BMI 40 kg/m2, respectively. For measuring the waist circumference, we used the non-elastic tape, without any pressure on the body, at the end of the natural exhalation, from the narrowest waist region with a precision of 0.5 cm. We determined the size of the hip circumference by using a strapless tape on the most prominent part that was marked, without imposing any pressure on the body of a person with an accuracy of 0.5 cm. The waist to hip ratio was calculated by dividing the waist circumference by the hip circumference. To decrease the measurement errors, all of the measurements were done by one person.

- Assessment of dietary intake

For evaluating the usual dietary intake of all participants over the last year, we used a reliable and validated semi-quantitative standard food frequency questionnaire with 147 food items [51]. Based on this questionnaire, the subjects were asked to report the frequency of their food consumption for each food item on a daily, weekly, monthly or yearly basis. During the face-to-face interview, the average size of each food item in the FFQ was explained to all individuals and participants were asked to rate the frequency of consumption of each food item according to their standard unit on a daily, weekly, monthly or annually. The information obtained from this questionnaire was entered into a file that was designed in excel program to determine the weight (grams) of each food item. The size of standard units and items reported on the basis of home scales were converted to grams using the home scale guide. Thus, the equivalent of consumption was obtained for each item and for each person. This converting and analyses down using the dietary intake data by using the NUTRITIONIST 4 (Hearst Corporation, San Bruno, CA) food analyzer. Total energy, macro and micronutrients were calculated by using Nutritionist 4 software (Hearst Corporation, San Bruno, CA) [52]. Major food groups in this study are cereals, simple sugars, red Meat, fish, meat of organs, fast food, chicken and eggs, low fat dairy products, high fat dairy products, fruits, dried fruit, natural juices, industrial juices and soft drinks, vegetables, junk food, beans, nuts, vegetables oils, unhealthy oils such as animal oils, mayonnaise, butter, and margarine., salt and salty foods like pickles, and pickled cucumbers.

- Assessment of IPAQ

To assess the level of physical activity (PA) of participants, we used an international physical activity questionnaire-short form (IPAQ). IPAQ includes the time and frequency of normal activities in each week of daily life during the past year. The level of physical activity of participants is expressed in metabolic equivalent hours per week (METs-h/week) [53].

#### Assessment of SMI

Skeletal muscle mass index (SMI) was calculated by following formula [54]:

ASM (kg) = lean body mass of extremity-bone mass of extremity.

SMI (%) = ASM (kg)/body weight (kg) × 100.

#### Assessment of DAL

Both net endogenous acid production (NEAP) and potential renal acid load (PRAL) have been proposed to explain dietary acid load (DAL) [10]. NEAP is considered a ratio of protein to potassium intake. PRAL is calculated using phosphorus, calcium, and magnesium in addition to protein and potassium. As a consequence, PRAL and all scores are better formulas to assess the acid load of the diet.

### Statistical analysis

At first, we categorized subjects according to PRAL and NEAP. Kolmogorov–Smirnov test, showed that all variables had normal distribution. To investigate continuous variables (including demographics, and lifestyle) across groups of dietary acid–base load indices. To investigate continuous variables (including demographics, and lifestyle) across groups of dietary acid–base load indices, one-way analysis of variance (ANOVA) was applied. A chi-square test was used to investigate the distribution of categorical variables (supplement use, educational status, job, income, and marriage) across groups of dietary acid–base load indices. Associations between dietary acid–base load indices and bone mineral content, skeletal muscle mass, and skeletal muscle mass index were examined using linear regression in different models. The following variables were considered for adjustment: energy intake (constant), age (constant), income (low, moderate, and high), physical activity (constant), supplement use (yes/no), marital status (married, single, and divorced), job (housekeeper/labor/management employee/non-managerial employee/household jobs, and university student), education (literate/primary education/intermediate education/high school education/diploma/postgraduate education, and bachelor's degree and higher. SPSS software was used to perform the statistical analysis (version 21.0; SPSS Inc, Chicago, IL). Statistical significance was accepted at p < 0.05.

## Results

### Study population and general characteristics

General and anthropometric variables of the study participants are reported in Tables 1 and 2. PRAL and NEAP have mean SDs of -16.85 ± 23.34 and 34.61 ± 9.79 (mEq/day), respectively. Among NEAP tertiles, a significant difference was found in age and physical activity (PA) (*P* < 0.05). This significance was also found in age, PA, and supplement use among PRAL tertiles. The mean SD of SMM and BMC were 25.45 ± 3.29 (kg) and 2.63 ± 0.35 (kg) respectively, and there was no significant difference between SMM and BMC across PRAL and NEAP tertiles.

**Table 1:** Characteristics of investigating subjects

Variable	Mean	SD	Minimum	Maximum
Age (years)	36.69	9.20	18.00	64.00
Body weight (Kg)	81.17	12.26	59.50	136.60
BMI (Kg/m^2^)	31.27	4.30	25.20	40.60
BMC (kg)	2.65	0.35	1.82	3.55
SMM (kg)	25.54	3.42	17.30	37.30
SMI (%)	31.67	3.21	25.18	50.75
IPAQ (METs-min/w)	1201.96	2103.80	49.50	19194.00
NEAP	34.61	9.79	7.24	75.46
PRAL	-16.85	23.34	-132.97	53.23
Energy (Kcal)	2633.281	809.43	1028.98	5157.99

*SD* Standard deviation, *BMI* Body mass index, *SMI* Skeletal muscle mass index, *IPAQ* International Physical Activity Questionnaires, *PRAL* Potential renal acid load, *NEAP* Net endogenous acid production, *BMC* Bone mineral content, *SMM* Skeletal muscle mass; met-min/w: metabolic equivalent-minute/week

**Table 2:** General characteristics of participants across two groups of NEAP and PRAL

**Variables**	**NEAP** (mEq/day)					**PRAL** (mEq/day)		
	T1(*n* = 130)	T2(*n* = 131)	T3(*n* = 130)	^†^*P*-value	T1(*n* = 130)	T2(*n* = 131)	T3(*n* = 130)	^†^*P*-value
**Education(n) %**								
Illiterate	(1)0.8	(1)0.8	(2)1.6	0.20	(1) 0.80	(1) 0.80	(2) 1.6	0.82
Primary education	(6)4.7	(3)2.3	(5)3.9		(5) 3.9	(4)3.1	(5)3.9	
intermediate Education	(12)9.3	(6)4.6	(8)6.2		(11)8.5	(8)6.1	(7)5.4	
High school education	(4)3.1	(4)3.1	(1)0.8		(4)3.1	(3)2.3	(2)1.6	
Diploma	(43)33.3	(50)38.2	(30)23.3		(47)36.4	(41)31.3	(35)27.1	
Postgraduate education	(11)8.5	(9)6.9	(8)6.2		(10)7.8	(10)7.6	(8)6.2	
Bachelor's degree and higher	(52)40.3	(58)44.3	(75)58.1		(51)39.5	(64)48.9	(70)54.3	
**Job(n) %**								
Housekeeper	(1)0.8	0	(1)0.8	0.20	(1)0.8	0	(1)0.8	0.32
Labor	(75)58.1	(83)63.8	(69)53.9		(77)59.7	(77)59.2	(73)57	
Management employee	(1)0.8	(3)2.3	0		(1)0.8	(3)2.3	0	
Non- managerial employee	(24)18.6	(16)12.3	(27)21.1		(20)15.5	(20)15.4	(27)21.1	
household jobs	(14)10.9	(18)13.8	(21)16.4		(15)11.6	(20)15.4	(18)14.1	
University student	(9)7	(5)3.8	(2)1.6		(10)7.8	(4)3.1	(2)1.6	
**Marriage(n) %**								
Married	(102)79.1	(89)67.9	(90)69.8	0.07	(95)73.6	(90)68.7	(96)74.4	0.67
Single	(22)17.1	(32)24.4	(37)28.7		(28)21.7	(32)24.4	(31)24	
Away from spouse more than 6 month	0	(3)2.3	0		(1)0.8	(2)1.5	0	
Dead spouse	(1)0.8	(2)1.5	0		(1)0.8	(2)1.5	0	
Divorce	(4)3.1	(5)3.8	(2)1.6		(4)3.1	(5)3.8	(2)1.6	
**Supplementation(n) %**								
Yes	(53)52	(49)41.9	(56)48.7	0.30	(55)53.9	(44)37.3	(59)51.8	**0.02**
No	(49)48	(68)58.1	(59)51.3		(47)46.1	(74)62.7	(55)48.2	
**Income(n) %**								
< 5,000,000 Rials	0	0	(1)0.9	0.60	0	0	(1)0.9	0.76
5,000,000–10,000,000 Rials	(3)3.1	(2)1.8	(1)0.9		(3)3.1	(2)1.8	(1)0.9	
15,000,000 Rials	(4)4.2	(4)3.5	(8)7		(3)3.1	(6)5.3	(7)6.2	
> 15,000,000 Rials	(36)37.5	(35)30.7	(40)34.8		(32)32.7	(42)36.8	(37)32.7	
*Age (y)	38.62 ± 8.91	36.43 ± 9.41	35.02 ± 9.00	**0.00**	38.21 ± 9.03	36.55 ± 9.30	35.32 ± 9.14	**0.04**
*BMI (kg/m^**2)**^	31.44 ± 4.30	31.53 ± 4.40	30.84 ± 4.20	0.37	31.61 ± 4.18	31.28 ± 4.43	30.93 ± 4.30	0.44
*PA (METs -min/w)	1854.97 ± 3516.16	1090.13 ± 1139.20	781.26 ± 735.11	**0.00**	1782.41 ± 3414.67	1236.12 ± 1636.93	768.43 ± 740.22	**0.00**
*Bone mineral content (BMC) (kg)	2.65 ± 0.34	2.70 ± 0.36	2.60 ± 0.33	0.08	2.67 ± 0.36	2.64 ± 0.33	2.63 ± 0.35	0.58
*Skeletal muscle mass (SMM) (kg)	25.48 ± 3.3	25.99 ± 3.65	25.15 ± 3.17	0.13	25.84 ± 3.48	25.33 ± 3.48	25.45 ± 3.29	0.45
*Weight (kg)	81.16 ± 12.89	82.92 ± 13.21	81.17 ± 12.26	0.07	82.15 ± 12.8	81.09 ± 12.96	80.28 ± 10.95	0.46

PRAL^1^: potential renal acid load; NEAP^2^: net endogenous acid production; BMI: body mass index; PA: physical activity, FFM: fat free mass, BFM: body fat mass, WHR: waist to hip ratio

Statistical significance was accepted at *p* < 0.05

^†^ Calculated by Chi-square and analysis of variance (*ANOVA*) for qualitative and quantitative variables, respectively

^*^Mean ± SD

### Dietary intake of macronutrient and food groups according to the PRAL and NEAP tertiles

The dietary intakes of participants are shown in Table 3. Among NEAP tertiles, calcium (*P* = 0.01), potassium (*P* = 0.00), protein (*P* = 0.00), magnesium (*P* = 0.00), carbohydrate (*P* = 0.00), and sodium (*P* = 0.04) were statistically significant after adjusting energy intake. In addition, calcium, potassium, magnesium, carbohydrate, fat, fiber, and all food groups' intake remained significant among PRAL tertiles (*P* < 0.05). Energy intake was also a significant difference among PRAL tertiles (*P* = 0.00).

**Table 3:** Energy-adjusted dietary intakes across two groups of NEAP and PRAL

*Variables		**NEAP** (mEq/day)				**PRAL** (mEq/day)		
	**T1(*n* = 130)**	**T2(*n* = 131)**	**T3(*n* = 130)**	^†^***P*-value**	**T1(*n* = 130)**	**T2(*n* = 131)**	**T3(*n* = 130)**	^†^***P*-value**
Energy (Kcal)	2663.13 ± 828.88	2709.06 ± 837.86	2527.07 ± 753.78	0.16	2889.06 ± 818.72	2567.89 ± 833.17	2443.40 ± 710.15	**0.00**
Calcium(mg/day)	1329.89 ± 463.38	1290.48 ± 398.38	1185.29 ± 311.62	**0.01**	1402.61 ± 515.12	1239.94 ± 301.03	1163.50 ± 310.32	**0.00**
Potassium (mg/day)	5430.88 ± 1111.66	4417.83 ± 829.56	3681.45 ± 752.69	**0.00**	5523.13 ± 1100.41	4363.54 ± 716.74	3643.92 ± 714.28	**0.00**
Protein(g/day)	87.43 ± 14.93	91.66 ± 16.63	94.83 ± 19.70	**0.00**	98.85 ± 31.17	88.53 ± 29.98	86.56 ± 32.03	0.52
Phosphorus (mg/day)	1663.32 ± 301.78	1704.37 ± 311.30	1656.78 ± 320.28	0.40	1700.86 ± 331.77	1663.97 ± 288.78	1659.96 ± 312.28	0.48
Magnesium (mg/day)	512.95 ± 109.33	483.36 ± 90.12	430.64 ± 83.72	**0.00**	528.60 ± 114.94	464.91 ± 76.70	433.58 ± 82.37	**0.00**
Carbohydrate(g/day)	389.49 ± 41.83	373.45 ± 41.43	354.40 ± 46.17	**0.00**	426.93 ± 126.26	363.12 ± 120.59	327.36 ± 105.84	**0.00**
Fat(g/day)	97.81 ± 16.39	94.18 ± 13.89	93.43 ± 17.91	**0.00**	99.38 ± 34.81	92.67 ± 36.07	93.38 ± 34.48	**0.00**
Fiber(g/day)	50.53 ± 22.38	47.68 ± 19.40	43.80 ± 21.81	0.06	56.19 ± 22.22	45.46 ± 20.17	40.38 ± 18.54	**0.00**
Sodium(mg/day)	4251.65 ± 1198.59	4644.43 ± 1363.07	4550.93 ± 1407.87	**0.04**	4662.96 ± 1831.47	4474.02 ± 1634.12	4311.34 ± 1798.01	0.14
**Food groups**								
Grains	424.97 ± 182.89	500.24 ± 208.44	520.68 ± 273.10	**0.00**	469.16 ± 214.64	468.94 ± 192.43	508.02 ± 269.32	**0.00**
Legumes				0.96	51.94 ± 42.08	44.48 ± 40.94	46.29 ± 36.01	**0.62**
Fruits	629.48 ± 368.67	467.35 ± 266.89	319.73 ± 238.58	**0.00**	647.51 ± 371.69	464.16 ± 245.97	304.91 ± 233.74	**0.00**
Vegetables	553.09 ± 291.67	408.88 ± 216.45	287.85 ± 197.43	**0.00**	576.19 ± 290.92	394.62 ± 201.68	279.12 ± 189.67	**0.00**
Red meat	21.68 ± 18.13	20.99 ± 22.81	22.36 ± 20.57	0.40	24.49 ± 24.26	20.97 ± 19.05	19.57 ± 17.61	**0.96**
Fish	9.42 ± 11.27	10.16 ± 9.09	13.30 ± 14.92	0.01	10.74 ± 12.47	9.98 ± 10.14	12.15 ± 13.41	**0.18**
Poultry	25.53 ± 19.91	34.22 ± 27.81	48.76 ± 54.24	**0.00**	30.53 ± 25.29	34.60 ± 30.91	43.38 ± 52.03	**0.00**
Dairy products	379.45 ± 231.51	400.45 ± 228.300	358.92 ± 276.67	0.77	410.32 ± 237.00	377.07 ± 221.41	351.56 ± 276.07	**0.93**

*PRAL*^1^ potential renal acid load, *NEAP*^2^ net endogenous acid production

All the variables, except energy, adjusted for energy intake

Statistical significance was accepted at *p* < 0.05

^*^Mean ± SD

^†^Calculated by analysis of variance (*ANOVA*)

### Association of NEAP and PERAL on the SMI, BMC, SMM among obese and overweight women subjects

As shown in Table 4, we had 3 models which were crude: model 1 (adjusted for age, PA, and energy) and model 2 (adjusted for age, PA, energy intake, education, job, marital status, supplementation use, and income status). A significant relationship between both PRAL and NEAP and SMI (skeletal muscle mass index) was reported in the crude model (*P* = 0.05). Linear regression revealed a continued negative relationship between NEAP and SMI in models 1 (β = -0.04, 95%CI = -0.096 to -0.001, *P* = 0.04) and 2 (β = -0.05, 95%CI = -0.097 to -0.003, *P* = 0.03). In models 1 (β = -0.02, 95%CI = -0.049 to -0.002, *P* = 0.03) and 2 (β = -0.02, 95%CI = -0.049 to -0.004, *P* = 0.02), SMI and PRAL were inversely related (β = -0.02, 95%CI = -0.049 to -0.004, *P* = 0.02). Other variables did not show any significant association between PRAL and NEAP among obese and overweight women.

**Table 4:** Association of NEAP and PERAL on the SMI, BMC, SMM among obese and overweight female subjects

		Β	95% CI	*P*-value
		**SMI (%)** ^a^		
NEAP (mEq/day)	Crude	-0.04	(-0.094, -0.001)	**0.04**
	*M1*	-0.04	(-0.096, -0.001)	**0.04**
	*M2*	-0.05	(-0.097, -0.003)	**0.03**
PRAL (mEq/day)	Crude	-0.02	(-0.045, -0.001)	**0.04**
	*M1*	-0.02	(-0.049, -0.002)	**0.03**
	*M2*	-0.02	(-0.049, -0.004)	**0.02**
		**BMC (kg)** ^a^		
NEAP (mEq/day)	Crude	-0.00	(-0.007, 0.004)	0.51
	*M1*	-0.00	(-0.008, 0.003)	0.32
	*M2*	0.00	(-0.007, 0.008)	0.92
PRAL (mEq/day)	Crude	-0.00	(-0.003, 0.002)	0.60
	*M1*	-0.00	(-0.004, 0.002)	0.49
	*M2*	-0.00	(-0.005, 0.002)	0.48
		**SMM (kg)**^a^		
NEAP (mEq/day)	Crude	-0.00	(-0.057, 0.046)	0.83
	*M1*	-0.00	(-0.059, 0.046)	0.81
	*M2*	0.00	(-0.08, 0.09)	0.97
PRAL (mEq/day)	Crude	0.00	(-0.24, 0.026)	0.93
	*M1*	0.00	(-0.023, 0.28)	0.82
	*M2*	0.00	(-0.017, 0.018)	0.94

M1: Adjusted for age, PA, energy intake

M2: Adjusted for age, PA, energy intake, education, job, marital status, supplementation use, income status and height

Statistical significance was accepted at *p* < 0.05

^a^Linear regression; *CI* confidence interval, *PERAL* Potential renal acid load, *NEAP* Net endogenous acid production, *SMI* Skeletal muscle mass index, *BMC* Bone mineral content, *SMM* Skeletal muscle mass

## Discussion

In the present study, we found an inverse association between SMI and both NEAP and PRAL in pre-menopause women with overweight or obesity. However, there was no significant association of DAL with muscle mass and BMC. In line with this study, a prospective cohort study, Chan et al. found that higher NEAP is associated with more muscle loss among 3122 older adults [22]. Many studies have shown an inverse effect of sodium intake on NEAP, as in the as present study [55]. Fruits and vegetables are known as the main sources of buffer in the diet due to their potassium content, which helps electro-neutrality through exchange with hydrogen ions in the distal part of the nephron [56]. The dietary amounts of potassium, magnesium, calcium, and carbohydrates decreased constantly across the increasing tertiles of NEAP and PRAL. They are related to more muscle mass and bone density [57, 58]. One study found the proper ratio of alkalinogenic to acidogenic foods: fruits and vegetables / intakes of meats, fish, eggs, dairy, and cereals to be 1/4 [25]. In line with this study, Ströhle et al. found no relationship between vegetable and fruit intakes and NEAP [59]. Baranauskas et al. found that for the optimal dietary acid–base balance and muscle adaptation to exercise, it is recommended that athletes consume higher amounts of potassium, magnesium, and calcium [60]. Animal sources produce acidic precursors due to incomplete oxidation, but vegetable proteins produce alkaline precursors in the body [61]. An imbalance between acidic and alkaline precursors has been shown to change the chronic net DAL. Delimaris discovered that it may have a negative impact on bone health [62] but this study did not agree with the statement. In line with this study, Mclean et al. found that higher PRAL and NEAP were not associated with BMC at any age of men or women, probably because of the protective effects of protein [63].

Contrary to Mohammadpour et al. study, the dietary amount of red meat, fish, poultry, and dairy products decreased constantly across the increasing PRAL tertiles [64]. Calder et al. found that excessive consumption of sulfur amino acids, which are widely found in animal foods, was associated with increased bone resorption [65]. Dietary methionine can decrease blood pH and increase musculoskeletal pain [66]. Metabolic acidosis can waste skeletal muscle through the ubiquitin–proteasome pathway and insulin-like growth factor-1 signaling [43]. Also, the participants were obese, non-menopausal women, and we know that obesity and estrogen may have protective effects on BMC [67, 68]. This might explain the insignificant association between NEAP and PRAL and BMC. On the other hand, for BMC estimates, dual-energy X-ray absorptiometry (DXA) is the gold standard method, and based on previous studies, BIA methodology is less reliable for measuring BMC because of some limitations [69]. This could be a reason of the insignificance result between BMC and PRAL and NEAP.

We found a weak association between SMI and DAL. However, we did not find any association with SMM. These different results may be due to the fact that SMI takes into account the height and muscle mass of an individual [70]. The association between DAL and SMI is less clear. Indeed, it is possible that higher DAL had a marginal effect on muscle mass index or that the association between DAL and muscle mass index may depend on population characteristics, which needs to be investigated by further studies. Welch et al. suggest that lower DAL is associated with greater SMI in 2,689 women aged 18–79 years from the Twins UK Study [25]. Overall, shifting toward plant-based products like nuts, oils, grains, soy, etc. instead of acid producing foods may protect skeletal muscle mass of the population and it can be a useful suggestion for improving the musculoskeletal health [71].

Choosing premenopausal women can be one of the strengths of this study and adjust this confounder due to the protective effect of estrogen on muscle health [72]. These novel findings suggest that a diverse and balanced diet, specifically one with higher consumption of fruit and vegetable may be important in having a high SMI [73]. Our study was a cross-sectional study, which means we could not draw a causal conclusion. Although we controlled for most lifestyle factors and diet quality, residual or unmeasured confounding factors cannot be excluded due to the study's observational nature. We used FFQ to assess dietary intake and measurement errors such as underreporting of dietary intakes are inevitable. According to some previous studies, BIA may overestimate the lean body mass when compared to the dual energy X-ray absorptiometry (DEXA) method [74, 75]. Measuring BMC through a more accurate method is recommended. Finally, some factors including small sample size, highly educated participants and absence of males in this study limit the extrapolation of our findings to other populations with different characteristics.

## Conclusion

The findings provide important information about the negative association between SMI and both NEAP and PRAL among overweight and obese women. No association was found between more aciditic PRAL and NEAP with BMC and SMM. Further research is needed to explore the extent of DAL and musculoskeletal health.

## Data Availability

The datasets used and/or analyzed during the current study are available from the Khadijeh Mirzaei on reasonable request.

## Abbreviations

BMI: Body mass index
DAL: Dietary acid load
NEAP: Net endogenous acid production
PRAL: Potential renal acid load
BMD: Bone mineral density
BMC: Bone mineral content
SMI: Skeletal muscle mass
FFQ: Food frequency questionnaires
WC: Waist circumference
WHR: Waist to hip ratio
IPAQ: International Physical Activity Questionnaire
ELISA: Enzyme linked immunosorbent assay
PA: Physical activity
